# Planning for a pinniped response during a marine oil spill

**DOI:** 10.1007/s11356-025-36192-y

**Published:** 2025-04-04

**Authors:** A. A. Hall, B. L. Chilvers, J. S. Weir

**Affiliations:** 1 Wildbase, School of Veterinary Science, Massey University, Private Bag 11 222, Palmerston North, 4442 New Zealand; 2 Department of Conservation, 115 Ludstone Rd, Kaikōura, 7300 New Zealand

**Keywords:** Fur seals, Pinniped, Oil spill response, *Arctocephalus forsteri*, Abundance, Hazing

## Abstract

Understanding the distribution and abundance of wildlife populations is key to successful oil spill response planning. Fur seals are difficult to rehabilitate if oiled, and many common spill response techniques may be limited in the high-energy rocky shore habitats they prefer. Preventing oil from reaching colonies, and hazing or deterring animals away from oil are high-priority response options for pinnipeds during spills. To do this, local knowledge of pinniped distribution and abundance is required, as well as knowledge of effective and safe hazing and deterrence mechanisms. From pup production assessments, we estimated that a population of 13,147–17,675 New Zealand fur seal (NZFS: *Arctocephalus forsteri*) currently inhabits Banks Peninsula. This area contains the largest port on New Zealand’s South Island and a secondary port that is popular with cruise ships, elevating its oil spill risk profile. From the knowledge gained regarding NZFS distribution and abundance, we evaluated mitigation methods which could protect fur seals during oil spills, wherever these species occur, and make suggestions to managers on how to mount an effective pinniped response.

## Introduction

Acute oil spills are among the most highly publicised threats to marine environments and the animals that exist within them (Fan et al. 2015). Given the many deleterious impacts of marine oil spills, and public expectations of a response to oiled wildlife (Safford et al. 2012; Henkel and Ziccardi 2018), many countries now require companies involved in oil acquisition and transportation to produce response plans that include consideration of oiled wildlife (Chilvers et al. 2021). In such plans, the optimum wildlife response is preventing the oil from reaching animals (Wolfaardt et al. 2008; Nijkamp et al. 2014; Ziccardi et al. 2015; Hong et al. 2020). This necessitates rapid mobilisation of personnel and resources and requires that baseline ecological data are in place (Battershill et al. 2016) regarding what species are likely to be present, where they are likely to be, and their local abundance. Even if the prevention objective cannot be met, knowledge of species’ pre-spill distribution and abundance is key to determining the incident’s ecological impact and informing subsequent management (Lewis et al. 2020; Fraser et al. 2022).

New Zealand’s most significant oil spill was the grounding of the *MV Rena* off Tauranga in 2011 (Schiel et al. 2016), with an estimated minimum of ca. 2,500 seabirds, 24 terrestrial birds, 17 kekeno/New Zealand fur seal (‘NZFS’; *Arctocephalus forsteri*) and three whales oiled as a result (Sievwright 2014). One of the recommendations derived from studies of this incident was that ecological data should be collected for areas where spills are most likely to occur in New Zealand, such as near ports and drilling platforms (Battershill et al. 2016).

**Fig. 1:**
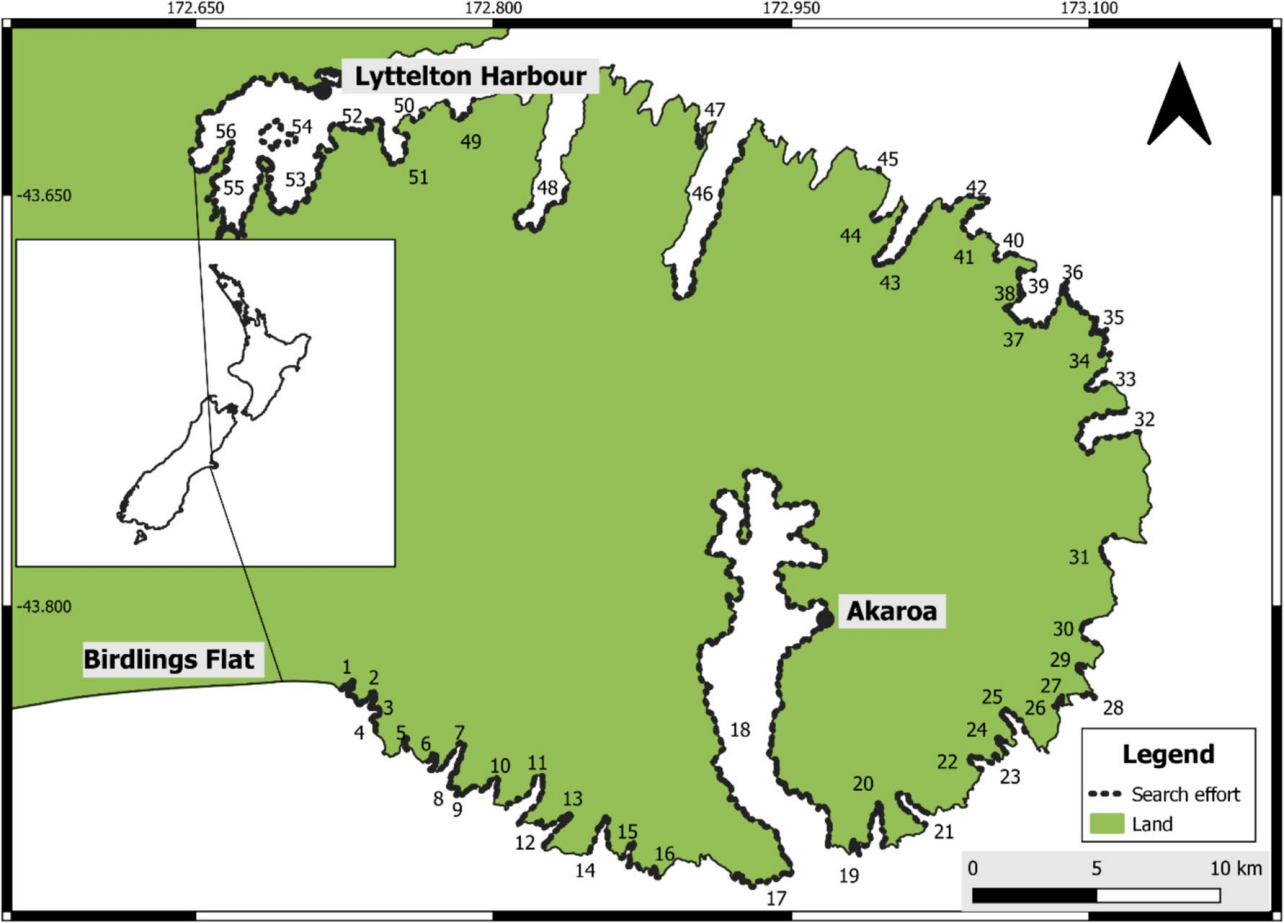
Key locations within the study area and distribution of search effort. The names of the bays are shown as numbers and correspond as follows: 1. Oashore Bay, 2. Tokoroa Bay, 3. Hikuraki Bay, 4. Magnet Bay beach, 5. Murrays Mistake, 6. Tumbledown Bay, 7. Te Oka Bay, 8. Te Kaio Bay, 9. Hells Gate, 10. Robin Hood Bay, 11. Peraki Bay, 12. Unnamed Bay, 13. Horseshoe Bay, 14. Long Bay, 15. Island Bay, 16. Whakamoa Bay, 17. Scenery Nook, 18. Akaroa Harbour, 19. Haylocks Bay, 20. Damons Bay, 21. Pōhatu Bay, 22. Stony Bay, 23. Reef Nook, 24. Sleepy Bay, 25. Otanerito Bay, 26. Red Bay, 27. Shell Bay, 28. Goat Point, 29. Paua Bay, 30. Goughs Bay, 31. Hickory Bay, 32. Le Bons Bay, 33. Lavericks Bay, 34. Ducksfoot Bay, 35. Pā Bay, 36. East Head, 37. Okains Bay, 38. Spyglass Point, 39. North West Bay, 40. Stony Bay, 41. Raupo Bay, 42. Long Lookout Point, 43. Little Akaloa Bay, 44. Decanter Bay, 45.South of Squally Bay, 46. Pigeon Bay, 47. Little Pigeon Bay, 48. Port Levy/Koukourarata, 49. Camp Bay, 50. Pile Bay, 51. Purau Bay, 52. Diamond Harbour, 53. Charteris Bay, 54. Otamahua/Quail Island, 55. Head of the Bay, 56. Governors Bay. All artwork created in QGIS 3.26.0 (QGIS.org, 2024)

Maritime NZ, the government authority responsible for responding to significant oil spills in New Zealand, identified Lyttelton Harbour, Banks Peninsula (Fig. 1), as falling into a high-risk classification category for oil spills in New Zealand (Navigatus Consulting 2015). Lyttelton is the South Island’s major port, and one of the busiest in the country (Inglis et al. 2008). Further, the recent closure of New Zealand’s only oil refinery means the country is now totally reliant on overseas oil imports (Wilson et al. 2023), and important ports such as Lyttelton will experience greater numbers of oil transportation vessels, heightening local spill risk (Chilvers et al. 2021). Banks Peninsula is an ecologically important area, home to threatened, endangered and rare species including Hector’s dolphins (*Cephalorhynchus hectori*) (Carome et al. 2022) spotted shags (*Stictocarbo punctatus*) (Brough et al. 2019), white flippered penguins (*Eudyptula minor albosignata*) (Allen et al. 2011), as well as NZFS (Ryan et al. 1997; Bradshaw et al. 1999; Boren et al. 2006). The risk of oil spills around Banks Peninsula is heightened by the proximity of a second port at Akaroa (Fig. 1). Akaroa is not a commercial port but is popular with large cruise boats (Carome et al. 2022), and Banks Peninsula is also important for commercial fishing. Shipping is the most common source for acute marine oil spills (Chilvers et al. 2021), meaning increased vessel traffic raises the overall risk profile for Banks Peninsula.

Fur seals (the nine *Arctocephalus* spp. and the northern fur seal (*Callorhinus ursinus*)) are at particular risk from oil spills due to their biology and life history (Mearns et al. 1999; Helm et al. 2015). Fur seals rely on their fur for insulation, and oil fouling significantly hinders their thermoregulatory capabilities (Mearns et al. 1999; Helm et al. 2015), reducing their ability to swim and forage (Ziccardi et al. 2015). This contrasts with all other pinnipeds, including phocids (true seals), odobenids (walrus), and sea lions which rely on blubber for insulation and so do not have the same dense, hard-to-clean fur (Ziccardi et al. 2015). The typical high density of fur seal colonies also means that proximal oil spills have the capacity to impact large numbers of animals at once, demonstrated by the deaths of at least 4,738 South American fur seal (*Arctocephalus australis*) pups following the *San Jorge* spill off Uruguay (Mearns et al. 1999). Large mammals like fur seals are also highly challenging to rescue and rehabilitate (Northwest Area Committee 2019). Only certain age classes can often be considered for treatment if oiled, as the size and aggressiveness of sub-adults and adult (particularly males) means that response options beyond monitoring are likely to be limited (Chilvers 2017). The *San Jorge* spill demonstrated the difficulty of treating even the smallest fur seals age cohorts, as all 41 pups taken for cleaning ultimately died (Mearns et al. 1999). Except for New Zealand sea lion (*Phocarctos hookeri*) pups, and potentially some juveniles or small females of that species, there would be limited ability to treat other, transient pinnipeds that occur in New Zealand – southern elephant seals (*Mirounga leonina*) due to their size, and leopard seals (*Hydrurga leptonyx*) due to their aggressiveness. New Zealand also lacks permanent rehabilitation facilities capable of treating large numbers of oiled seals. The only facility in New Zealand that currently holds pinnipeds in captivity is Auckland Zoo, and transporting NZFS large distances to this centre, near the top of the North Island, would be impractical. As such, in New Zealand, like many countries, other response approaches, primarily aimed at preventing pinnipeds from becoming oiled, need to be explored and prioritised.

Despite the need for baseline ecological data in high spill risk areas (Battershill et al. 2016), NZFS’ high oiling likelihood (Chilvers 2017) and the presence of two ports on Banks Peninsula, there are no reliable contemporary distribution and abundance data for NZFS in this region. This is representative of much of New Zealand, where NZFS population monitoring has been ad hoc with regards to regularity and methodological approach (Dix 1993; Taylor et al. 1995; Boren et al. 2006; Bouma et al. 2008; Gooday et al. 2018; Chilvers 2021), as the species continues to recolonise areas within its former range post historic sealing (Dussex et al. 2016). Currently, no reliable nationwide abundance estimate for NZFS exists, with a figure of 200,000 used for the combined population of NZFS in New Zealand and Australia for over 20 years (Harcourt 2001; Goldsworthy and Gales 2008).

The recovery of fur seal species from historic exploitation has been observed in several countries (Kirkwood et al. 2010; Milano et al. 2020) and has often followed a documented pattern (Roux 1987), knowledge of which can inform oil spill response planning (see Discussion). On Banks Peninsula, the first post-sealing record of NZFS breeding was from Horseshoe Bay in 1973 (Wilson 1981), and the most recent thorough assessment of individual colonies was ca. 20 years ago, when pup production estimates of ca. 300 were calculated for Horseshoe Bay and Te Oka Bay (Boren et al. 2006).

This study aims to provide current understandings of NZFS abundance and breeding distribution on Banks Peninsula to facilitate regional oil spill response planning and species management. Based on the findings, we also discuss and evaluate possible mitigation and remediation options, to improve future pinniped responses during marine oil spills. These recommendations can be adapted for any country where fur seals are present.

## Materials and methods

Banks Peninsula is a peninsula of ca. 1150 square kilometres, located centrally on the east coast of New Zealand’s South Island (Fig. 1).

Locations of large aggregations of NZFS around Banks Peninsula were initially noted during land and boat-based surveys in October 2023. NZFS colonies on Banks Peninsula were determined through pup direct count and mark-recapture surveys between January 30th and February 29th, 2024. Pup production estimates are the most reliable indicators for total pinniped population sizes (Berkson and DeMaster 1985), and late January–March represents the period when the entire year’s cohort of NZFS pups will have been born, and thus are available to count, and still retain their instantly recognisable black natal pelage (Berkson and DeMaster 1985). Mark-recapture and direct counts of pups have been used to provide NZFS abundance estimates for several decades (Taylor et al. 1995; Bradshaw et al. 2003; Boren et al. 2006; Roberts and Neale 2016). Here, a ‘colony’ refers to a breeding aggregation of NZFS, after Shaughnessy et al. (2015).

Most surveys were conducted from the land, but five sites (Fig. 1: Nos. 11 (west arm), 17, 18, 21, 32) (Fig. 1) were assessed from vessels. Survey effort distribution is shown in Fig. 1.

The methodologies for conducting direct counts and mark-recapture at NZFS colonies are well documented, and the protocols followed here mirror those described in recent studies in New Zealand (Chilvers 2021; Hall et al. 2024). Notably, in some locations on Banks Peninsula where access to breeding was unfeasible or unsafe, direct counts were conducted from a vantage point such as a clifftop. (Table 1).

**Table 1:** Numbers of New Zealand fur seal pups marked at sites on Banks Peninsula

	Pups marked	
Site	Number	Percentage of site total (%)
Te Oka Bay – east	70	20.47
Horseshoe Bay – east	60	15.74
Whakamoa Bay	65	25.49
Otanerito Bay – north	37	16.9
Goat Point	35	28.46
Total	267	

From visual recapture surveys, the ratio of marked to unmarked pups in each recapture sample was input into a modified Petersen pup production estimate (Chapman 1952). Full descriptions of the calculations are provided in Gales and Fletcher (1999).

As direct counts are known to produce underestimates (Watson et al. 2009), wherever mark-recapture was undertaken a direct count was also conducted so that calibration indices could be calculated. These were used to convert the results from direct count only sites into results comparable with the mark-recapture results (Table 2) (Watson et al. 2009; Chilvers 2021; Hall et al. 2024).

**Table 2:** Comparison of direct count and mark-recapture results at sites where both were conducted to show derivation of calibration indices

Site	Direct count result	Mark-recapture result	Calibration index
Te Oka Bay – east	186	333	1.80
Horseshoe Bay – east	185	377	2.04
Whakamoa Bay	194	255	1.31
Otanerito – north	115	215	1.88
Goat Point	107	124	1.16

Two estimates of pup production were derived from these analyses. A minimum pup production estimate was calculated by summing the mark-recapture results and the direct counts only sites. Second, a converted pup estimate was calculated by multiplying the results of direct count only sites by a calibration index and adding these results to the sum of the mark-recapture estimates.

Multipliers have previously been used to convert pup production estimates into population estimates (Shaughnessy et al. 1994, 1995; Campbell et al. 2014; Chilvers 2021; Hall et al. 2024). Here, Goldsworthy and Page’s (2007) multiplication factor of 4.76 was used.

In New Zealand, oil spill responses are coordinated by either the regional council in the impacted area, or at a national level by Maritime New Zealand (Maritime NZ), depending on the spill size. There is also a National Oiled Wildlife Response Team run out of Massey University, with trained responders located throughout the country. Maritime NZ publishes a periodically reviewed Oil Spill Readiness and Response Strategy (Maritime NZ 2021), which details the systems and procedures in place for responding to major oil spills that go beyond the capacity of regional councils. Regional councils create similar documents for coordinating responses in their regions. Environment Canterbury (ECAN) is the regional council responsible for responding to oil spills impacting Banks Peninsula. If a spill were to occur around Banks Peninsula, depending on its exact location, the closest available spill kits are in Akaroa or Lyttelton, and there are approximately 30 people trained to respond to a spill in the region (Environment Canterbury 2023).

## Results

**Fig. 2:**
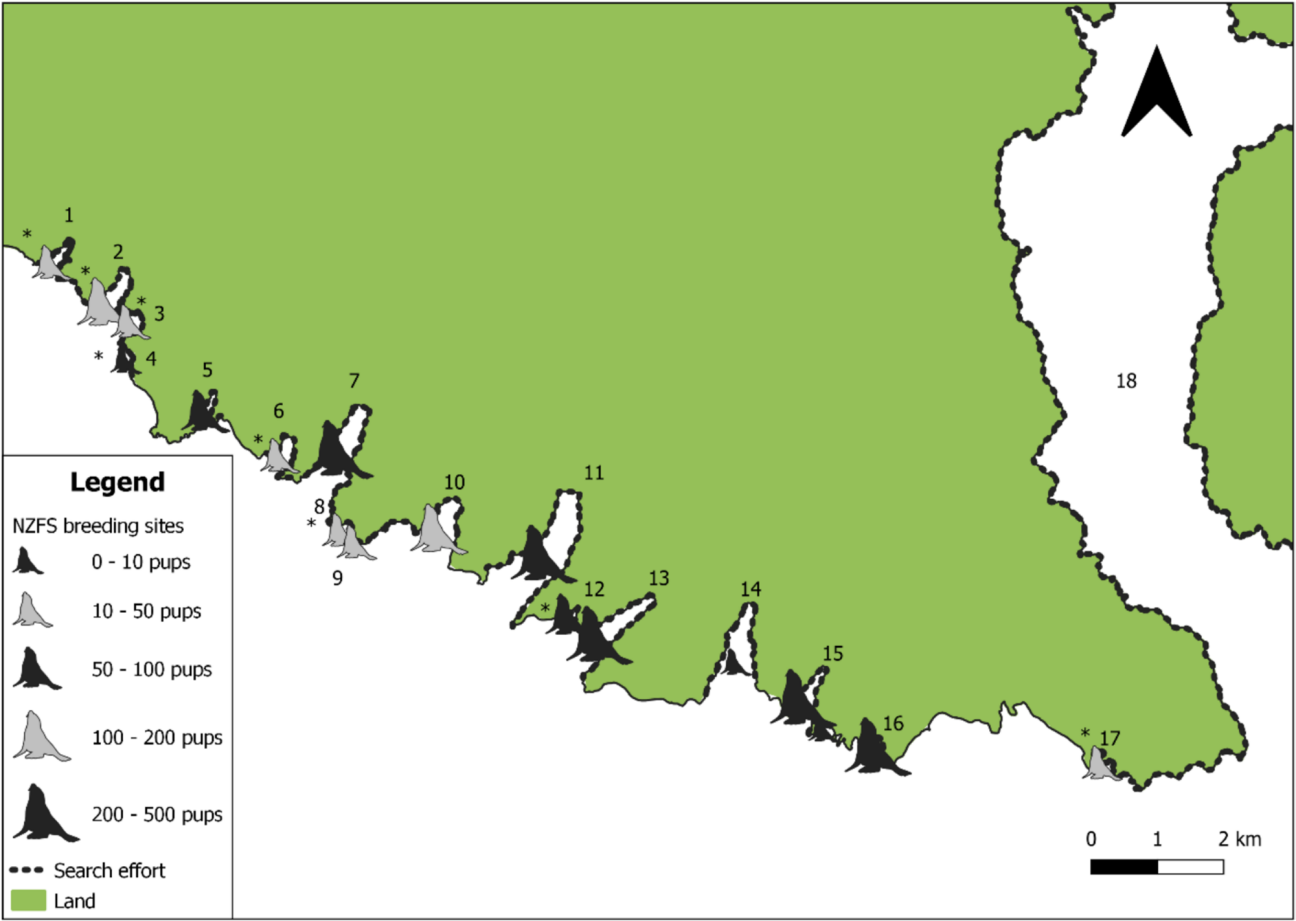
Locations of NZFS colonies on the south coast of Banks Peninsula. Colonies marked with asterisk (*) are sites where there is no previous record of NZFS breeding (Baird 2011; Emami-Khoyi et al. 2016). The names of the bays are shown as numbers and correspond as follows: 1. Oashore Bay, 2. Tokoroa Bay, 3. Hikuraki Bay, 4. Magnet Bay beach, 5. Murrays Mistake, 6. Tumbledown Bay, 7. Te Oka Bay, 8. Te Kaio Bay, 9. Hells Gate, 10. Robin Hood Bay, 11. Peraki Bay, 12. Unnamed Bay, 13. Horseshoe Bay, 14. Long Bay, 15. Island Bay, 16. Whakamoa Bay, 17. Scenery Nook, 18. Akaroa Harbour

**Fig. 3:**
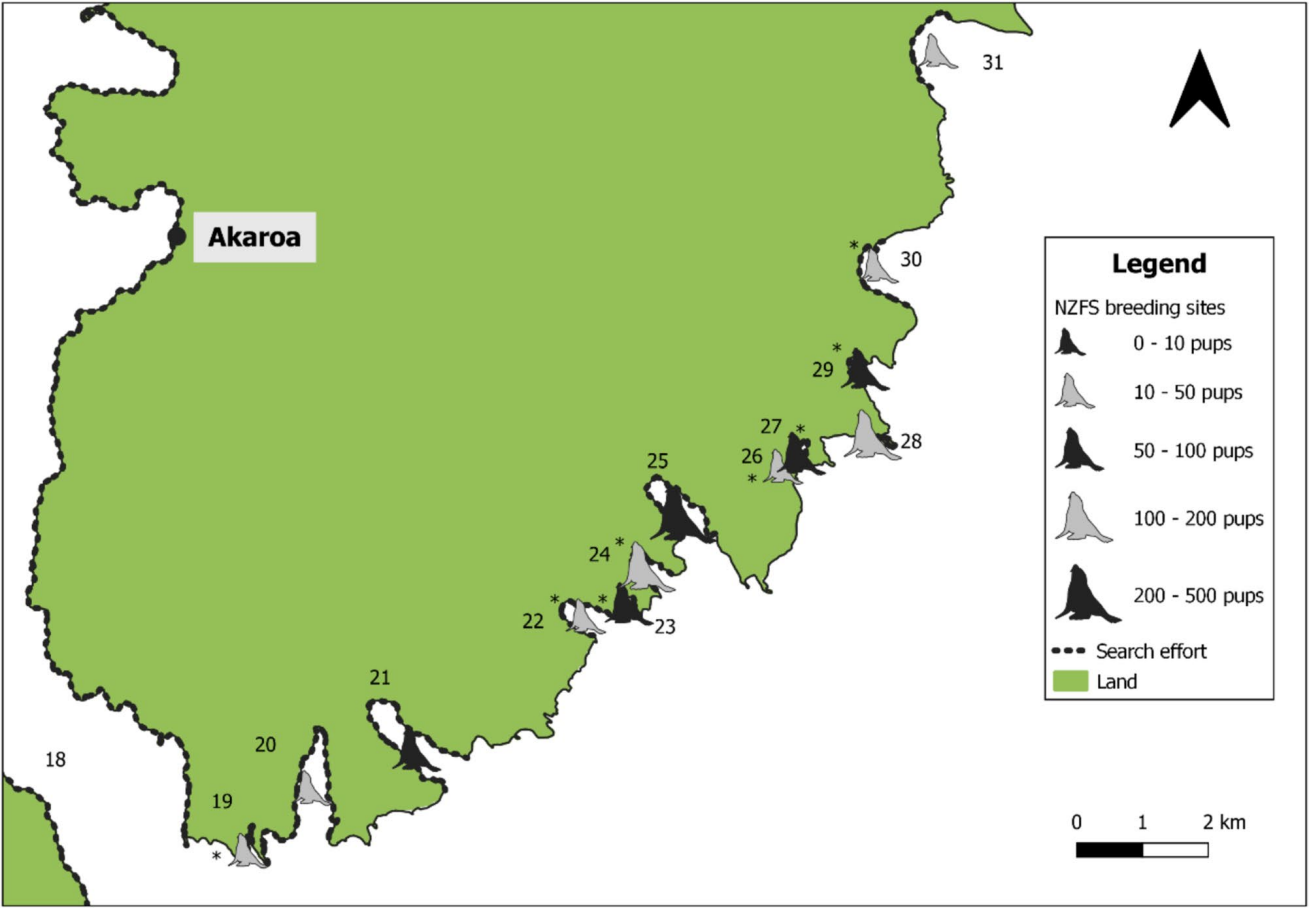
Locations of NZFS colonies on the south-eastern coast of Banks Peninsula. Colonies marked with asterisk (*) are sites where there is no previous record of NZFS breeding (Baird 2011; Emami-Khoyi et al. 2016). The names of the bays are shown as numbers and correspond as follows: 18. Akaroa Harbour, 19. Haylocks Bay, 20. Damons Bay, 21. Pōhatu Bay, 22. Stony Bay, 23. Reef Nook, 24. Sleepy Bay, 25. Otanerito Bay, 26. Red Bay, 27. Shell Bay, 28. Goat Point, 29. Paua Bay, 30. Goughs Bay, 31. Hickory Bay

**Fig. 4:**
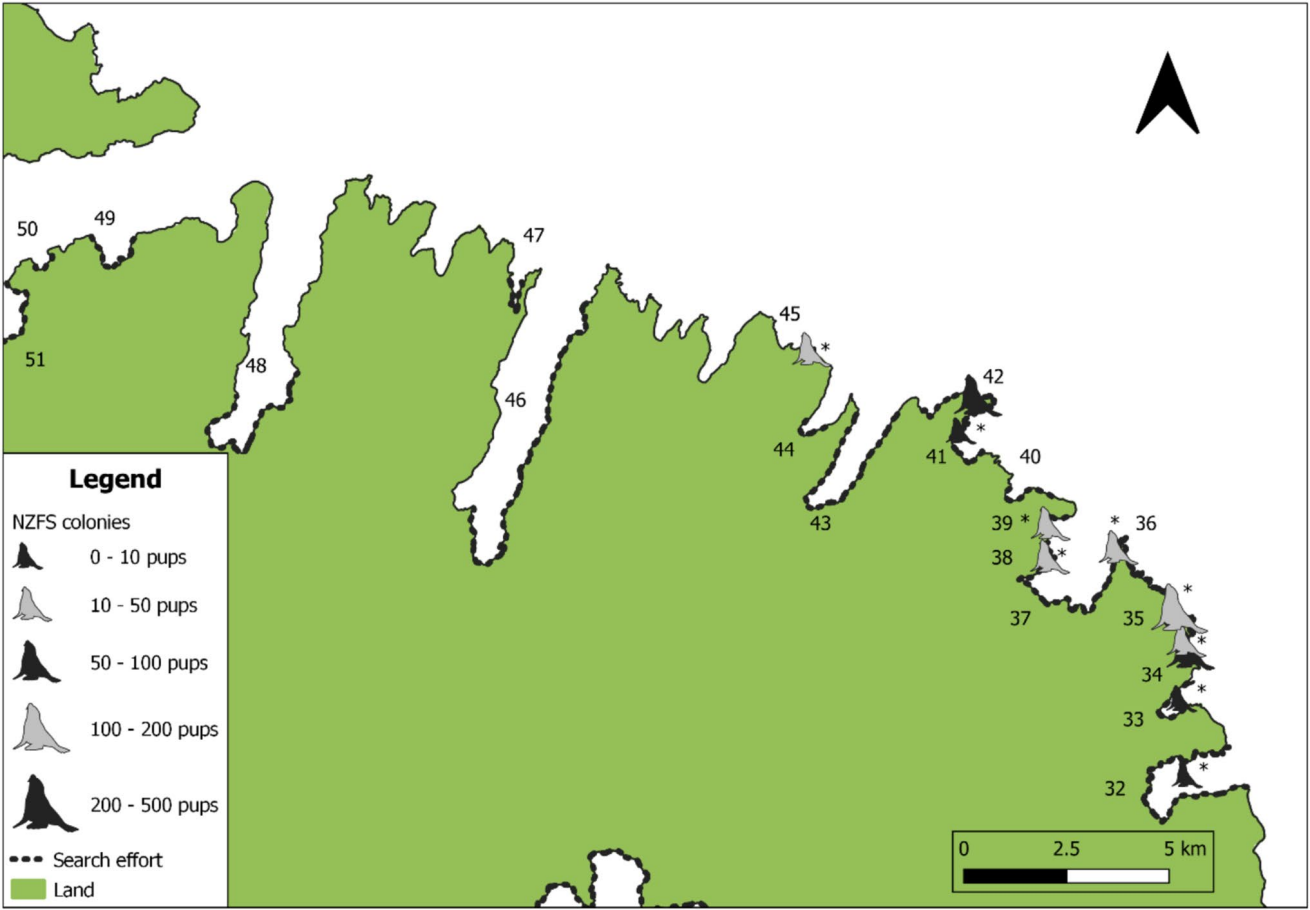
Locations of NZFS colonies on the north-eastern coast of Banks Peninsula. Colonies marked with asterisk (*) are sites where there is no previous record of NZFS breeding (Baird 2011; Emami-Khoyi et al. 2016). The names of the bays are shown as numbers and correspond as follows: 32. Le Bons Bay, 33. Lavericks Bay, 34. Ducksfoot Bay, 35. Pā Bay, 36. East Head, 37. Okains Bay, 38. Spyglass Point, 39. North West Bay, 40. Stony Bay, 41. Raupo Bay, 42. Long Lookout Point, 43. Little Akaloa Bay, 44. Decanter Bay, 45.Squally Bay, 46. Pigeon Bay, 47. Little Pigeon Bay, 48. Port Levy/Koukourarata, 49. Camp Bay, 50. Pile Bay, 51. Purau Bay

The distribution of NZFS breeding around Banks Peninsula is shown in Figs. 2, 3, and 4. A total of 41 colonies were identified, 25 of which had not been surveyed before. South of Okains Bay (Figs. 1 and 4), pupping occurred at all searched sites other than Akaroa Harbour, and the headland directly south-west of the Akaroa Harbour mouth. NZFS pupping north of Okains Bay was less consistent relative to search effort. No NZFS pups were recorded west of Squally Bay on the north coast of Banks Peninsula, or within Akaroa Harbour (Figs. 1 and 4).

A total of 267 pups were marked during mark-recapture across five sites (Table 1), producing an estimated pup production of 1,321 (± 15 SE). Direct count only sites (Table 3) produced a total estimated pup production of 1451 (± 2 SE). The results from the mark-recapture sites and direct-count only sites were summed to produce a minimum NZFS pup production estimate for Banks Peninsula of 2,762–2,774 pups in the 2023/24 breeding season.

**Table 3:** New Zealand fur seal pup production estimates for Banks Peninsula in the 2023/24 breeding season

Site	Colony reference no. (Figs. 1, 2, 3 and 4)	Pup direct count (mean ± SE)	Pup mark-recapture estimate (mean ± SE)	Converted bay total (mean ± SE)
Oashore^*^	1	27		48
Tokoroa^*^	2	84 ± 3		151 ± 3
Hikuraki^*^	3	10 ± 1		18 ± 1
Magnet Bay Beach^*^	4	1		2
Murray’s Mistake^*^	5	46 ± 3		83 ± 3
Tumbledown^*^	6	17		31
Te Oka – west^*^	7	42 ± 1		418 ± 1
Te Oka – east		195 ± 8	342	
Te Kaio^*^	8	10		18
Hells Gate^*^	9	14		26
Robin Hood Bay^**^	10	60 ± 1		123 ± 1
Peraki Bay^**^	11	153 ± 5		312 ± 5
Unnamed bay between Peraki and Horseshoe^**^	12	47 ± 2		96 ± 2
Horseshoe Bay – west^**^	13	51 ± 1		485 ± 20
Horseshoe Bay – east		189 ± 14	381 ± 18	
Long Bay^**^	14	1		2
Island Bay^**^	15	101 ± 5		206 ± 5
Headland between Island Bay and Whakamoa Bay^***^	Between 15 and 16	6 ± 2		7 ± 2
Whakamoa Bay – west^***^	16	99 ± 14	119 ± 5	255 ± 12
Whakamoa Bay – east^***^		95 ± 9	135 ± 11	
Scenery Nook^***^	17	30		39
Haylocks Bay^***^	19	12		16
Damons Bay^****^	20	14		26
Pōhatu Bay^****^	21	45		85
Stony Bay^****^	22	15 ± 1		28 ± 1
Reef Nook^****^	23	28 ± 1		53 ± 1
Sleepy Bay^****^	24	70 ± 1		132 ± 1
Otanerito Bay – south ^****^	25	12 ± 1		241 ± 16
Otanerito Bay – north		119 ± 3	219 ± 9	
Red Bay^****^	26	15 ± 1		28 ± 1
Shell Bay^****^	27	37 ± 2		69 ± 2
Goat Point^*****^	28	107 ± 7	124 ± 4	124 ± 4
Paua Bay^*****^	29	68 ± 6		79 ± 6
Goughs Bay^*****^	30	12 ± 1		14 ± 1
Hickory Bay^*****^	31	28 ± 2		32 ± 2
Le Bons Bay^*****^	32	9		10
Lavericks Bay^*****^	33	6		7
Ducksfoot Bay^*****^	34	84 ± 1		97 ± 1
Headland between Ducksfoot Bay and Pā Bay^*****^	Between 34 and 35	18		21
Pā Bay^*****^	35	130 ± 4		150 ± 4
Okains Bay – East Head^*****^	36	25 ± 2		29 ± 2
Okains Bay – Spyglass Point^*****^	38	32 ± 1		37 ± 1
North West Bay^*****^	39	14		16
Raupo Bay^*****^	41	7		8
Long Lookout Point^*****^	42	65 ± 1		75 ± 1
East of Squally Bay^*****^	Between 44 and 45	10		12
Total		**1,451 ± 2**^**a**^	**1,321 ± 15**	
Minimum pup estimate ^b^		**2,762 – 2,774**		
Converted pup estimate ^c^			**3,702 – 3,713**	
Coarse estimated total population range (multiplier 4.76) ^d^		**13,147 – 17,675**		

Empty cells indicate no count of that type was conducted in that location

^a^This total is the sum of the direct counts for the sites where mark–recapture was not also undertaken

^b^The minimum pup estimate is the sum of the direct counts for the sites where mark–recapture was not undertaken, and the mark-recapture results from the remaining sites

^c^The converted pup estimate is the sum of the mark-recapture estimates combined with the sum of the products of each direct count multiplied by the given calibration index

^d^This population estimate equals the range between the lower bound of the minimum pup estimate multiplied by 4.76 and the upper bound of the converted pup estimate multiplied by 4.76 (Goldsworthy & Page 2007)

^*^Te Oka Bay multiplier used to convert direct counts

^**^Horseshoe Bay multiplier used to convert direct counts

^***^Whakamoa Bay multiplier used to convert direct counts

^****^Otanerito Bay multiplier used to convert direct counts

^*****^Goat Point multiplier used to convert direct counts

Five calibration indices were calculated from the sites where both mark-recapture and direct counts were conducted (Table 2).

Five indices were calculated as Banks Peninsula demonstrates substantial variation in its topography, making a single calibration index unlikely to be appropriate for use at all sites (Watson et al. 2009). Calibration index selection was based on matching most closely the physical characteristics of the bay in question with one for which a calibration index existed (Table 3). When direct count only sites were calibrated and added to the mark-recapture estimates, the converted pup production estimate for Banks Peninsula was 3,702–3,713.

By subjecting the lower bound of the minimum pup production estimate (2,762) and the upper bound of the converted pup production estimate (3,713) to Goldsworthy and Page’s (2007) 4.76 multiplier (Chilvers 2021; Hall et al. 2024), a total NZFS population estimate for Banks Peninsula in the 2023/24 breeding season was calculated as 13,147–17,675.

## Discussion

Knowledge of species distribution and abundance is important not only for oil spill response planning (Battershill et al. 2016), but also for determining the short and long-term ecological impacts of oil spills (Lewis et al. 2020; Fraser et al. 2022), or other unexpected events (Hall et al. 2024). This study sought to provide important population parameters for NZFS on Banks Peninsula, in the first comprehensive survey of the region. In the 2023/24 breeding season, NZFS breeding was identified at 41 bays or headlands around Banks Peninsula, 25 of which had not previously been recorded (Figs. 2, 3, and 4; Table 3). Using a combination of mark-recapture and direct counts, a minimum pup production estimate of 2,762–2,774 was produced. As direct counts typically produce underestimates (Watson et al. 2009), calibration indices were used to produce a more reliable pup production estimate of 3,702–3,713. The total NZFS population on Banks Peninsula in 2023/24 was estimated at 13,147–17,675. This figure is likely still an underestimate, as five colonies where small numbers of pups had previously been noted (Baird 2011) could not be accessed. The largest NZFS colonies on Banks Peninsula by pup production were Horseshoe Bay (485 ± 20 SE), Te Oka Bay (418 ± 1 SE), and Peraki Bay (312 ± 5 SE), all of which are on the peninsula’s southern coastline.

### Implications for pinniped responses during oil spills

The population and geographic expansion of any seal species in an area with a high oil spill risk classification (Navigatus Consulting 2015) is something that managers need to be aware of when planning efficient and successful responses to oil spills (Battershill et al. 2016). During a spill, the welfare of charismatic megafauna like NZFS would receive significant interest from public and the media (Paine et al. 1996), particularly as many fur seal species are the subjects of eco-tourism operations.

NZFS terrestrial distribution is not static through the year (Stirling 1971; Miller 1975; Mattlin 1978; Boren 2005), meaning that the timing of an oil spill should be considered when coordinating a response. Oil spills occurring near breeding colonies during NZFS’ austral summer breeding season (December–February) would likely carry the greatest risk of mass oiling, as this is when the densest NZFS aggregations form. In particular, in the days and weeks following peak pupping, newborn pups, adult males and adult females will all be present at breeding colonies, whereas later in the breeding season, many adult and sub-adult males leave the breeding colony and form separate bachelor colonies (Crawley and Wilson 1976), and as pups grow, females begin to extend the duration of their foraging trips away from colonies (Harcourt et al. 2002). The *San Jorge* incident off Uruguay demonstrated the devastation that can be caused by an oil spill proximal to a breeding colony during the breeding season, with nearly 5,000 South American fur seal pups dying (Mearns et al. 1999). The peak pupping date for NZFS at Banks Peninsula colonies is not known, and, in other regions, has been shown to vary both spatially and annually. For example, at Ōhau Point (Kaikōura), the median pupping date was December 16th in 2002 and December 5th in 2003 (Boren 2005), while two distinct colonies in Otago had median pupping dates of the 24th and 29th of December in 1993 (Lalas and Harcourt 1995). Notably, this study does not document the locations of bachelor colonies around Banks Peninsula, meaning that an oil spill occurring outside of the breeding season may impact NZFS ashore in locations other than those depicted in Figs. 2, 3, and 4. However, options for rescuing or rehabilitating adult or sub-adult male NZFS would be limited due to their size and aggression.

Preventing wildlife from becoming oiled should always be the primary objective in oil spill responses (Ziccardi et al. 2015; Hong et al. 2020), and is particularly important for fur seals. With other pinnipeds (e.g. phocids and sea lions), there may be greater opportunities for rescue and rehabilitation, as effective treatment would likely be less time intensive, due to these species’ relative lack of fur (Ziccardi et al. 2015). Although, again, it is likely that only certain age classes of other pinnipeds could be rescued, due to the sizes that adults of some species attain. Additionally, some phocids, such as leopard seals, ribbon seals (*Histriophoca fasciata*) and bearded seals (*Erignathus barbatus*) do not form large terrestrial aggregations, reducing their likelihood of mass oiling. Contrastingly, the difficulties associated with successfully rescuing and rehabilitating fur seals once oiled (Mearns et al. 1999; Ziccardi et al. 2015; Chilvers 2017; Northwest Area Committee 2019), and the potential for of mass oiling (Gales 1991; Mearns et al. 1999), mean that prevention, rather than rescue and rehabilitation, should be the primary objective. There are typically three ways to achieve this goal: (1) containing the oil as quickly as possible, (2) diverting oil away from habitats, (3) deterring or hazing wildlife from areas with spilled oil (IPIECA 2014; Chilvers and McClelland 2023; Chilvers 2024).

Various methods exist for containing, diverting or removing spilled oil. These include physical barriers (booming) (Ghaly and Dave 2011), in situ oil burn offs (Fritt-Rasmussen et al. 2023), chemical dispersants to degrade oil droplets and assist natural weathering (Prince 2015), and bioremediation (Okeke et al. 2022). Each of these methodologies has strengths and weaknesses (Mullin and Champ 2003; Yang et al. 2009; Ghaly and Dave 2011; Dimitrakiev et al. 2020), including limitations based on local environmental conditions (Yang et al. 2009; Ghaly and Dave 2011), regulations and response practices. For example, in situ burning is often avoided close to coastlines due to concerns for human health and the risk of secondary fires (Ghaly and Dave 2011; Fritt-Rasmussen et al. 2023), and is not permitted within New Zealand waters.

On Banks Peninsula, several common remediation technologies are unlikely to be viable and/or desirable for protecting NZFS from oil spills. For example, dispersants can be toxic to marine taxa (Muncaster et al. 2016) and can cause loss of waterproofing in birds, replicating the impacts of oil itself (Whitmer et al. 2018). Additionally, booming is unlikely to work along much of the southern and south-eastern coastlines of Banks Peninsula, where the densest NZFS colonies are found (Figs. 2, 3, and 4), due to high wave action, which can wash oil over these barriers (Ghaly and Dave 2011). This reality is likely to be repeated in other fur seal habitats, as these species mainly inhabit rocky shores, which are typically high energy. Sites on Banks Peninsula where booms could be deployed include Haylocks Bay (Fig. 3; No. 19), where NZFS pups are mostly at the head of this very narrow bay, away from swells, or Red Bay and Shell Bay (Fig. 3; Nos. 26–27) which are less directly exposed. Even at these sites, however, booming viability would depend on ocean and weather conditions during a spill. Booms could also be used effectively to contain oil spilled within Lyttelton and Akaroa Harbours. The waters in these areas are often relatively calm and, at Lyttelton in particular, the port entry is narrow and could be effectively sealed.

While ports are particularly likely to experience oil spills (Battershill et al. 2016), the grounding of the *Austro Carina*, a 25-m-long fishing vessel, on Banks Peninsula near Red Bay (Fig. 1, No. 26), in September 2023, served as a reminder that oil spills can occur anywhere vessels travel. Despite carrying 10,000 l of diesel and 400 l of hydraulic oil (Radio New Zealand 2023), and grounding near NZFS and spotted shag colonies, dedicated wildlife reconnaissance surveys by a trained biologist following the incident found no evidence of oiled wildlife, most likely because the grounding occurred at a time of year when fewer animals are typically present. Difficulties with deploying common oil containment and diversion equipment around Banks Peninsula mean that preventing or deterring NZFS from accessing impacted areas likely represent more effective mitigation options. Such approaches fall into two broad categories–pre-emptive capture (Chilvers and McClelland 2023) and deterrence/hazing (Chilvers 2024). With NZFS, both techniques would likely be required, given the differences in behaviour and habitat-use across NZFS at different ontological stages (Crawley and Wilson 1976). For oil spill response planning, the most salient difference is between pre-weaned pups (hereafter, ‘pups’), which are largely confined to their natal colony (Berkson and DeMaster 1985), and the remaining weaned individuals (hereafter, ‘non-pups’) which forage in the ocean and come ashore for rest and, at certain times of the year, breeding and pupping (McNab and Crawley 1975; Crawley and Wilson 1976).

Pups typically remain in their natal colony while their mothers are away foraging (McNab and Crawley 1975), meaning the former would be unlikely to vacate the coastline if oil was present, which could lead to mass oiling (Gales 1991; Mearns et al. 1999). In accessible colonies with low pup numbers, like Te Kaio (Fig. 2; No. 8), Red Bay (Fig. 3; No. 26) or Lavericks Bay (Fig. 4; No. 33), pre-emptive capture of pups (and any non-pups that can be safely contained) may be viable (Chilvers and McClelland 2023). NZFS pups fast while their mothers are away foraging (Harcourt et al. 2002), and could be contained for a few days in situ without feeding, at most times of the year, provided their health was monitored. However, this could not be performed for more than a few days due to a risk of starvation, and this period would be shorter in the first weeks of a pup’s life, when they suckle more regularly, meaning that mother–pup separation is more likely to lead to starvation. Ideally, as many non-pups as safely possible would be contained ashore too, as individuals oiled at sea returning to the colony would likely oil the terrestrial habitat and animals they encounter while ashore. Pre-emptive capture could allow time for oil to be removed from coastal substrates (Wolfaardt et al. 2008) or from the ocean (Ghaly and Dave 2011). However, this strategy would be logistically challenging and resource and personnel intensive (Chilvers and McClelland 2023), meaning it could only be maintained for a few days. As such, the emphasis should remain on promptly containing oil movement offshore, and removing oil from the ocean surface through mechanisms such as booms and skimmers (Ghaly and Dave 2011) and bioremediation (Okeke et al. 2022).

During spills, non-pups risk oil exposure both on land and at sea. The only viable option for sub-adult and adult males is monitoring, as their size would limit safe capture and handling, and even attempting to rescue and rehabilitate smaller non-pup females would likely be difficult (Chilvers 2017). Therefore, the best option for preventing oiling of non-pups would likely involve attempting to haze or deter them from areas where oil was present. Hazing wildlife involves using negative stimuli to move them out of an area, while deterrence uses unpleasant or fearful stimuli to engender an escape or avoidance response to prevent wildlife from entering an area (Chilvers 2024). Such methods have been used to keep pinnipeds away from fishing operations (Lehtonen et al. 2022), prevent predation on endangered fish species (Tidwell et al. 2021) and deter them from potentially harmful underwater sound sources (Mikkelsen et al. 2017), with mixed results. Numbers of Steller sea lions (*Eumetopias jubatus*) preying on endangered salmon on the Columbia River were reduced during hazing involving seal bombs (underwater pyrotechnics), paintballs, rubber bullets and cracker shells; however, sea lion behaviour returned to normal once hazing ceased (Tidwell et al. 2021). Additionally, avoidance responses declined over time, suggesting habituation (Tidwell et al. 2021). Mixed success has also been reported with acoustic harassment devices (AHDs), which proved successful in a study of grey seals (*Halichoerus grypus*) (Vetemaa et al. 2021) but not in another involving harbour seals (*Phoca vitulina*) (Mikkelsen et al. 2017).

Non-pups could easily be hazed off oil-impacted shorelines, as these individuals typically flee to the water when humans approach (pers. obs.). However, this may not be desirable if oil remained in the water around the colony, and, as such, should only be considered in the latter stages of a response, when water-borne oil has been contained or removed, and the intention is to clean fouled shorelines (Mearns et al. 1999). More practicable would be to haze or deter NZFS away from oil slicks. This would likely be achieved through a combination of pinniped hazing and deterrence devices such as AHDs and pyrotechnics (Mikkelsen et al. 2017; Tidwell et al. 2021; Vetemaa et al. 2021). It is advised that trials of such devices on NZFS should be performed prior to their deployment during an oil spill to assess their efficacy and evaluate potential impacts on other species. For example, there are concerns that seal bombs can cause temporary or permanent hearing loss in both pinnipeds and cetaceans (Simonis et al. 2020), the latter of which react to audio deterrence mechanisms at greater distances (Mikkelsen et al. 2017). The presence of Hector’s dolphins around Banks Peninsula (Carome et al. 2022), as well as transitory cetaceans (Gibbs et al. 2018) means that the impacts of hazing and deterrence on other species must be considered. Similarly, the presence of human activity, even during deterrence operations, can, in fact, attract curious and intelligent marine mammals (Wright et al. 2022), again emphasising the need for trials of such devices prior to their deployment on Banks Peninsula.

Regardless of methodology, effective deterrence or hazing would likely need to be continuous, and varied, to avoid NZFS habituation, for the duration of the risk to NZFS (Tidwell et al. 2021). Success would also depend on where the oil was relative to NZFS foraging grounds. At sea, NZFS are mostly either foraging or travelling to or between foraging sites (Page et al. 2005). Lactating female NZFS show fidelity both to their foraging sites, and the approximate routes taken from their colonies (Baylis et al. 2012). Given this site fidelity, and the fact that it is difficult to discourage animals away from easily accessible and abundant resources (Simonis et al. 2020; Tidwell et al. 2021), it may be harder to deter or haze NZFS from spilled oil within their foraging grounds, compared to oil enroute to these foraging grounds. Foraging ecology studies of Banks Peninsula’s NZFS, like those conducted in Australia (Page et al. 2005; Baylis et al. 2012), would complement the current study in identifying high-use habitats for local NZFS, and thus aid timely oil spill responses. Currently, it is assumed that Banks Peninsula’s NZFS forage over the continental shelf and shelf edge (Allum and Maddigan 2012), but the precise locations of foraging grounds are unknown. Once foraging grounds were located it would be useful to know whether hazing and deterrence device efficacy differed between foraging and non-foraging sites, to promote efficient resource use during a spill. Implementing the type of deterrence or hazing programs described above around Banks Peninsula would likely involve the deployment and coordination of significant numbers of personnel and other resources, such as vessels and equipment.

Given the size of Banks Peninsula, and the fact that local marine oil spill risks only derive from vessels, as opposed to, for example, wells, it is unlikely that there would be a spill so large that there were no un-oiled waters to haze animals towards, which can be a consideration in other regions (Ziccardi et al. 2015). From the results of this study, an oil spill threatening the southern coastline of Banks Peninsula would be the most concerning, as this is where the largest concentrations of NZFS are (Fig. 2). Notably, the southern side of the Peninsula experiences the heaviest seas, which, as noted, limits oil spill response options, but also means that oil is likely to weather more quickly.

While a pinniped response during a marine oil spill would be complex at Banks Peninsula, it would likely be less logistically difficult than if such an event occurred in lower Fiordland, where a similar sized NZFS population (13,971–24,000) was recently surveyed (Chilvers 2021). Fiordland is another area popular with cruise tourism, and its extreme remote location, often poor weather conditions and challenging terrain (Egan et al. 2023) could impede timely wildlife responses. More challenging again would be a spill impacting one of New Zealand’s subantarctic islands, which are also visited by cruise boats, and where some NZFS populations have not been monitored in ca. 30–45 years. Increases in global cruise tourism in recent decades, combined with trends towards cruise vessels visiting remote and/or ecologically important areas (Cerveny et al. 2020; Lau et al. 2023), as well as expansions of commercial shipping (Lau et al. 2023) make oil spills in such logistically challenging environments more likely (Chilvers et al. 2021). As such, it is important both that ecological data are collected for these areas, and that area-specific response plans are in place. In remote regions, this planning should include selecting optimum locations for housing response equipment, as well as safe and efficient access routes for response personnel. Additionally, comparatively accessible locations, such as parts of Banks Peninsula, as well as requiring their own response plans, offer the opportunity to trial response equipment, for example hazing apparatus, for use with populations of conspecifics in remote areas.

### Changes in NZFS abundance and distribution

Twenty-five of the 41 NZFS colonies identified in this study had not been previously recorded. Both the distribution and abundance of NZFS have increased when compared with previous surveys on Banks Peninsula (Ryan et al. 1997; Bradshaw et al. 1999; Boren et al. 2006), with the spread of colonies appearing to follow Roux’ (1987) description of fur seal recolonisation patterns, and Bradshaw’s (2000) suggestion that new colonies are founded close to existing ones in a density-induced spillover effect. This pattern has previously been suggested for Banks Peninsula (Boren 2005; Emami-Khoyi et al. 2016) and appears to have continued. The two colonies for which previous comparable data existed have both grown, but only slightly. Te Oka Bay (Fig. 2, No. 7) increased from ~ 300 pups in 2005 (Boren et al. 2006) to 418 (± 1 SE) pups in 2024, while Horseshoe Bay (Fig. 2, No. 13) grew from ~ 300 pups in 2005 (Boren et al. 2006) to 485 (± 20 SE) pups in 2024. These relatively small increases relative to other NZFS colonies in New Zealand (Hall et al. 2024), combined with the founding of new colonies, again suggests spillover (Bradshaw et al. 2000), while the substantial geographic expansion of NZFS breeding on Banks Peninsula highlights the need for regular population surveys to inform oil spill response plans. This regularity is particularly important in regions where pinnipeds are recolonising (Kirkwood et al. 2010; Milano et al. 2020), in order to ensure that plans’ ecological bases still reflect reality. In areas where fur seal population monitoring has not occurred for some time, knowledge of the spillover pattern of breeding distribution could help inform the search and recovery phase of an oil spill wildlife response (Hunter et al. 2019). Suitable, but previously unused, habitat close to known colonies may have become occupied since monitoring last occurred (Bradshaw et al. 2000; Hall et al. 2024), and thus could be prioritised when searching for oiled animals. However, it is preferable that regular population monitoring is conducted to ensure that the ecological foundations of oiled wildlife response plans remain current for a given area (Battershill et al. 2016).

In 2023/24, more pups were produced on the southern coastline relative to the remainder of Banks Peninsula, and no breeding was recorded west of Squally Bay on the north coast (Fig. 4). There are also no NZFS colonies within Akaroa or Lyttelton Harbours. There are several potential explanations for this. Generally, NZFS are thought to be recolonising New Zealand from South to North (Dussex et al. 2016), meaning that initial recolonisation (Wilson 1981) and subsequent spillover likely favoured Banks Peninsula’s south coast. Additionally, a lack of preferred terrestrial habitat features (Ryan et al. 1997; Bradshaw et al. 1999) in some northern bays and Akaroa Harbour, and/or the amount of vessel traffic in Lyttelton and Akaroa Harbours (Boren et al. 2002; Cowling et al. 2014) may have deterred NZFS colony foundation. These absences are also important to understand for planning oil spill responses. Ports are among the places where oil spills are most likely to occur (Battershill et al. 2016), but the absence of NZFS colonies in the immediate vicinity of both Lyttelton and Akaroa Harbours (Figs. 2, 3, and 4) means that, initially, NZFS swimming in the ocean nearby are more likely to be impacted than individuals ashore. If oil cannot be contained within these ports, the emphasis should be on modelling the likely direction the oil slick will move, in order to understand which, if any colonies, could be impacted. This will aid in the timely deployment of resources and personnel to threatened locations. Simultaneously, hazing or deterrence should be employed from the water to prevent NZFS from swimming into the slick.

A regular monitoring program is suggested for Banks Peninsula’s NZFS to enable timely detection of changes to population trends and allow managers to plan oil spill responses and respond to other concerns. The timing of this should be coordinated with other sites around the country to enable both spatial and temporal comparisons in population trajectories. Another benefit of regular monitoring is the potential to use the large, high-trophic level NZFS as sentinels for marine ecosystem change (Moore 2008; Bossart 2011), including for anthropogenic pollution (Brock et al. 2013; Donahoe et al. 2014; Taylor et al. 2018; Fulham et al. 2018) and climate change (Elorriaga-Verplancken et al. 2016).

## Conclusion

This study confirms an expanding NZFS population in an area of New Zealand with a high risk of oil spills (Navigatus Consulting 2015). The NZFS population estimate of 13,147–17,675 calculated here is considerably higher than any previous estimate for Banks Peninsula’s NZFS (Wilson 1981; M. Morrissey 2007, unpublished data; Baird 2011), and new colonies have been founded beyond those already recorded (Ryan et al. 1997; Boren 2005; Baird 2011). Recovering NZFS populations around New Zealand, and pinniped populations in other countries (Kirkwood et al. 2010; Milano et al. 2020), mean there are likely to be greater incidences of deleterious interactions between these species and human infrastructure and activities, including oil acquisition and transport facilities. As such, thorough and regularly reviewed plans need to be in place to inform oiled pinniped responses.

Given the potentially catastrophic consequences of oil spills for fur seals (Mearns et al. 1999; Ziccardi et al. 2015), preventing spilled oil from reaching land, and hazing or deterring pinnipeds out of slicks, should be prioritised by responders during a marine oil spill. To prepare, trials should be conducted to determine the efficacy of hazing and deterrence methods on NZFS, as no such studies have previously been conducted with this species. Additional areas of study that would be beneficial to oil spill responders, and in the general management of Banks Peninsula’s NZFS, include regular future monitoring of their distribution and abundance, and foraging ecology studies to ascertain the locations of favoured feeding grounds for individuals within this population.

## Data Availability

The datasets analysed during the current study are available from the corresponding author on reasonable request.
